# Impact of plant-derived antioxidants on heart aging: a mechanistic outlook

**DOI:** 10.3389/fphar.2025.1524584

**Published:** 2025-03-21

**Authors:** Muneer Ahmed Khoso, Heng Liu, Tong Zhao, Wenjie Zhao, Qiang Huang, Zeqi Sun, Khuzin Dinislam, Chen Chen, Lingyi Kong, Yong Zhang, Xin Liu

**Affiliations:** ^1^ State Key Laboratory of Frigid Zone Cardiovascular Diseases (SKLFZCD), Department of Pharmacology, College of Pharmacy, Department of Cardiology, The Second Affiliated Hospital, Harbin Medical University, Harbin, China; ^2^ State Key Laboratory-Province Key Laboratories of Biomedicine-Pharmaceutics of China, Key Laboratory of Cardiovascular Research, Ministry of Education, College of Pharmacy, Harbin, China; ^3^ Research Unit of Noninfectious Chronic Diseases in Frigid Zone (2019RU070), Chinese Academy of Medical Sciences, Harbin, China

**Keywords:** heart aging, cardiac alterations, antioxidants, mechanism, reactive oxygen species

## Abstract

Heart aging involves a complex interplay of genetic and environmental influences, leading to a gradual deterioration of cardiovascular integrity and function. Age-related physiological changes, including ventricular hypertrophy, diastolic dysfunction, myocardial fibrosis, increased arterial stiffness, and endothelial dysfunction, are influenced by key mechanisms like autophagy, inflammation, and oxidative stress. This review aims to explore the therapeutic potential of plant-derived bioactive antioxidants in mitigating heart aging. These compounds, often rich in polyphenols, flavonoids, and other phytochemicals, exhibit notable antioxidant, anti-inflammatory, and cardioprotective properties. These substances have intricate cardioprotective properties, including the ability to scavenge ROS, enhance endogenous antioxidant defenses, regulate signaling pathways, and impede fibrosis and inflammation-promoting processes. By focusing on key molecular mechanisms linked to cardiac aging, antioxidants produced from plants provide significant promise to reduce age-related cardiovascular decline and improve general heart health. Through a comprehensive analysis of preclinical and clinical studies, this work highlights the mechanisms associated with heart aging and the promising effects of plant-derived antioxidants. The findings may helpful for researchers in identifying specific molecules with therapeutic and preventive potential for aging heart.

## 1 Introduction

There is a growing interest in the field of medicine and aging research regarding the effects of plant-derived antioxidants on heart aging. The aging global population underscores the need of to understand the mechanism of heart aging mechanisms and exploring novel treatment techniques. Recent studies have focused on the potential of plant-based antioxidants to enhance cardiovascular health and mitigate the effects of heart aging (Phu et al., 2020; Zhang et al., 2022). The risk of cardiovascular diseases (CVDs), including heart failure, peripheral vascular disease, coronary heart disease, and stroke, is well recognized to increase with age (Flora and Nayak, 2019). Heart aging leads to alterations in heart structure and function, lowering the threshold for CVDs. With the projected increase in the aged population, nearly 50% of adult Americans are expected to have CVDs by 2030, resluting in greater burden of CVDs (Benjamin et al., 2017). The foremost cause of mortality in United States CVDs, are considerably increased by the aging process. A substantial proportion of CVDs, related fatalities and cases approximately 485·6 million are attributed to adults aged 75 years and above on a global scale (Zhou et al., 2016; Fan et al., 2023). Over 40% of all fatalities in China are attributed to CVD, making it the principal cause of mortality. Additionally, China has largest elderly population (aged 65 and above) in the world (Madhavan et al., 2018; Zhou et al., 2019). The exponential and steady growth of this demographic has presented significant challenges in the prevention and management of CVD (Slivnick and Lampert, 2019; Healthcare Engineering, 2022). In the past, efforts to reduce the risk of CVDs primarily focused on controlling known risk factors, which include hypertension, hyperglycemia, hyperlipidemia, and elevated circulating triglycerides.

**FIGURE 1:**
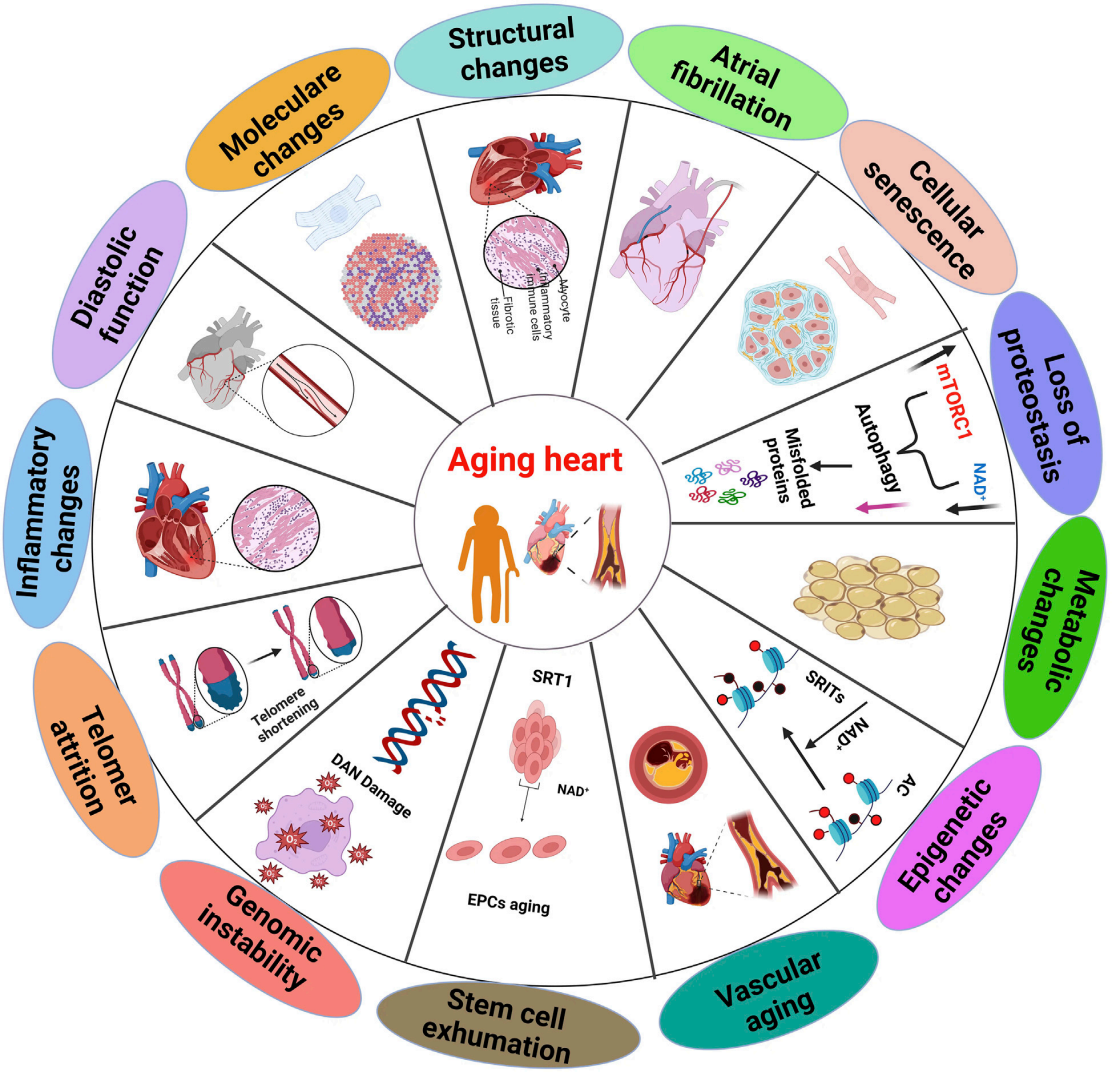
Multiple aspects of the aging heart, which displaying distinct sections and summary of the biochemical and physiological alterations that occur in the heart aging (Haddad et al., 2008).

Novel approaches to addressingCVDs, have been developed in recent preclinical studies. Research has shown that restricting calories reliably increase longevity in experimental animal models (Sciarretta et al., 2021). Calories restriction mimetic and other pharmaceutical interventions have been developed to delay the onset of age-related diseases, which are leading causes of mortality and morbidity due to their impact on the heart and blood circulation (Ma et al., 2020). Notable advancements in the field of aging studies have enhanced our understanding of the fundamental processes involved in aging, demonstrated that biological aging is adaptive (Saenjum et al., 2023). Age is a key risk factor for many persistent diseases that cause functional decline and loss of independence, such as dementia, type 2 diabetes, CVDs, and cancer, age is a key risk factor (Figure 1). With the substantial increase in the older adult population, projected to reach 12.6% in North America and 12% globally by 2030, it is expected that this percentage will continue to increase (Sinclair et al., 2020; Olayem et al., 2024).

## 2 Alterations related to heart aging

The aging process involves alterations in the intricate regulatory interactions between cells, organs, and systems (Guo et al., 2022). Cardiac and smooth muscle cells are involved in the involuntary regulation of heart and vascular activities (Chen et al., 2023). The cardiovascular regulation depends on the integrity, excitability, conductivity, contractility, and flexibility of these cells. Cellular aging associated with gradual decline in the physiological activity of cardiomyocytes and vascular smooth muscle cells (Huang and Lei, 2023). Aging causes structural changes in the cardiovascular system, that can impair the function and flexibility of the heart and blood vessels (Ribeiro et al., 2023).

**FIGURE 2:**
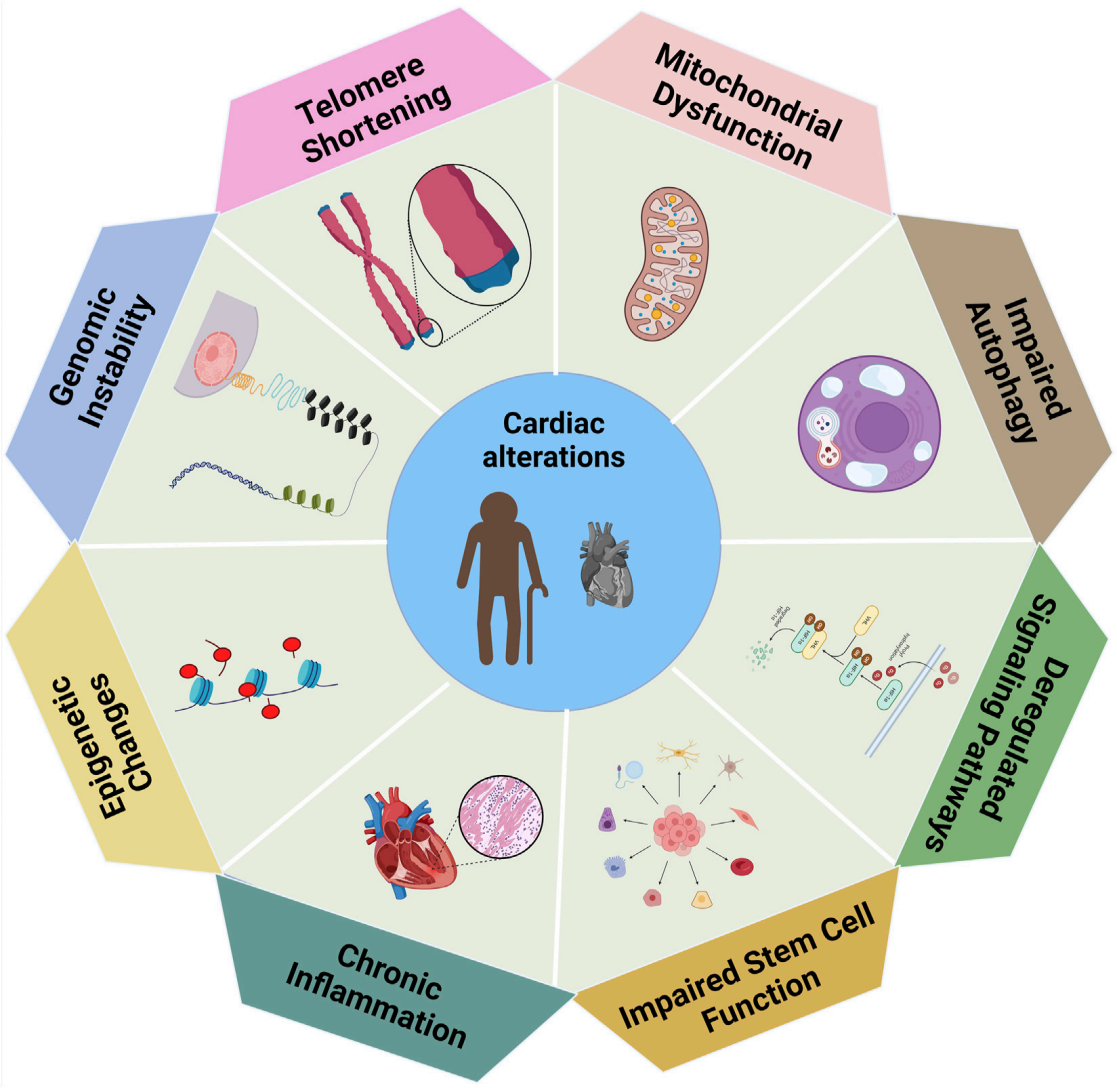
The biological variables associated with cardiac modifications, in which each element indicating a distinct contributor to changes in heart function (Handy et al., 2011).

In humans, heart aging is associated with an enlargement of left ventricle, development of fibrosis, and impaired ability of the heart to relax during diastole. This leads to reduced filling of the heart and decreased ejection fraction (Ribeiro et al., 2023; Hamo et al., 2024). While the precise mechanisms remain poorly understood, studies suggest that demise of cardiac myocytes is a crucial factor, (cardiomyocyte apoptosis) and increased stiffness of blood vessels are associated with the structural and functional changes that occur with aging (Anwar et al., 2024;Totoń-Żurańska et al., 2024). Aging significantly increases the susceptibility to numerous diseases. As a result, aging, the heart becomes predisposed to numerous detrimental structural and functional alterations as a result of aging (Figure 2), making age primary risk factor for CVDs. Generally, heart failure is most prevalent in the elderly population, particularly those over 65 years of age (Saheera and Krishnamurthy, 2020).

### 2.1 Heart hypertrophy and aging

The functional anomalies of the aged myocardium arise from both structural modifications and cellular and molecular changes. Heart aging is influenced by various molecular processes such as cardiac hypertrophy being a characteristic feature of this aging process in the heart (Hastings et al., 2024). As the heart ages, it undergoes hypertrophy, leading to changes in nutrient and growth signaling (Oldfield et al., 2020). The mechanistic target Rapamycin (mTOR) (Dai et al., 2023), and *insulin-like growth factor-1* (*IGF-1*) are two significant signaling pathways that have an involvement in heart hypertrophy and aging (Abdellatif et al., 2023). The mechanistic target of rapamycin (mTOR), governs cellular development and has been shown to be a significant regulator of the aging process and age related diseases (Liu and Sabatini, 2020). Enhanced *mTOR* signaling hinders resilience to heart aging, whereas diminished *mTOR* signaling improves it in mice models (Mannick and Lamming, 2023).

In animal models, the *insulin/IGF-1* signaling pathway is essential for controlling longevity. Deficiency in this signaling pathway in mice leads to a decrease in age-related dysfunction of cardiomyocytes (Lee and Kim, 2018). The decline in *IGF-1* levels associated with aging increases the heart failure risk in individuals (Khan et al., 2002). The functional damage caused by dysfunctional mitochondria can shorten life expectancy by impairing cellular and organ function (Amorim et al., 2022). Age-related diseases increase mitochondrial ROS production result in enlarged, bloated, and damaged mitochondria (Liu et al., 2020; Schneider et al., 2020). In the heart, mitochondrial function is regulated by *PGC-1α*, also known as peroxisome proliferator-activated receptor coactivator (Qian et al., 2024).

### 2.2 Cardiac dysfunction

Cardiac dysfunction results from the repression of its expression in failing hearts (He et al., 2022). Mitochondrial malfunction and aberrant ROS generation accelerate the aging process, by directly harming cellular macromolecules and interfering with normal regular signaling and energetics (Proshkina et al., 2020). The ECM, a conglomeration of proteins located outside cells, offers structural and metabolic support to neighboring cells (Karamanos et al., 2021). Cardiac fibroblasts are the main producers of ECM proteins, such as collagen, elastin, fibronectin, laminin, and fibrinogen (Halper, 2021). Heart stiffness increases when there is an abundance of ECM deposited on the heart contributed to diastolic dysfunction (Lunde et al., 2024). MMPs, TIMPs, and other proteases regulate the production and breakdown of ECM proteins (Shan et al., 2023). Aging hearts exhibit myocardial fibrosis and dysregulation of ECM protein production and breakdown. Profibrotic factors, such as transforming growth factor-β, stimulate the production of ECM proteins and hinder the breakdown of the matrix by MMPs (Antar et al., 2023; Lunde et al., 2024). The regulation of MMPs and TIMPs varies with on aging; however, their precise roles in heart aging remain inadequately understood (Rodrigues et al., 2024).

The process of heart aging causes microscopic alterations, such as an increase in the thickness of inner layer of blood vessels (intima) and the buildup of collagen. Symptoms includeelevated systolic blood pressure and pulse wave velocity, along with increase in pulse pressure (Xu et al., 2017; Liu W. et al., 2023). Left ventricular hypertrophy (LVH) may occur as a consequence of increased afterload and wall stress brought on by arterial stiffness. The volume of the myocardium remains constant with age in the heart (Đorđević et al., 2024). Research indicated that within the age range of 30–70 years, approximately 35% of the total myocytes in the ventricle are lost (Oknińska et al., 2022).

Tocounteract the loss of cells, the surviving myocytes undergo hypertrophy, accompanied by an increase in the nonmyocyte compartment. While the exact cause of cell death remains uncertain, aging is associated with a decrease in capillary density, which leads to ischemic injury, (Bradley et al., 2021). Mouse models are commonly used to study heart aging because they accurately replicate the characteristics of human heart aging (Lindsey et al., 2021). Another advantage of using mouse models to research the molecular underpinnings of heart aging is their relatively short lifespan and the availability of genetically engineered animals (García-García et al., 2021). Laboratory mice do not exhibit elevated blood pressure or adverse cholesterol and blood glucose levels, allowing researchers to study the natural heart aging process without additional cardiac complications (Badmus et al., 2023). The echocardiogram conducted on a mouse model revealed phenotypic alterations such as elevated left ventricular mass, impaired diastolic function, and deteriorated myocardial performance index (MPI), resembling the aging process of the human heart (Lazzeroni et al., 2022).

## 3 Oxidative stress

OS significantly contributes to aging and the development of degenerative and chronic diseases by binding to transition metal ions. This includes a range of conditions such as autoimmune disorders, inflammation, cancer, arthritis, neurodegenerative diseases, and cardiovascular issues (Leyane et al., 2022). It causes various health problems by triggering abnormalities (Lobo et al., 2010). It occurs when the ability of antioxidants to counteract pro-oxidant compounds is exceeded, OS occurs, leading to disruptions in biological signaling and pathological events, particularly in older adults (Martemucci et al., 2023). Antioxidants are vital for the body’s defense against oxidation, as they help prevent the formation of free radicals and minimize cellular damage (Bratovcic, 2020; Jomova et al., 2024). Non-enzymatic substances found in blood plasma, such as transferrin, ferritin, ceruloplasmin, and albumin, act as preventive antioxidants through attaching itself to ions of transition metals, thereby inhibiting the formation of new reactive species (Skoryk and Horila, 2023). These non-enzymatic antioxidants provide an intermediate defense, neutralizing oxidants and radicals (Rudenko et al., 2023).

**FIGURE 3:**
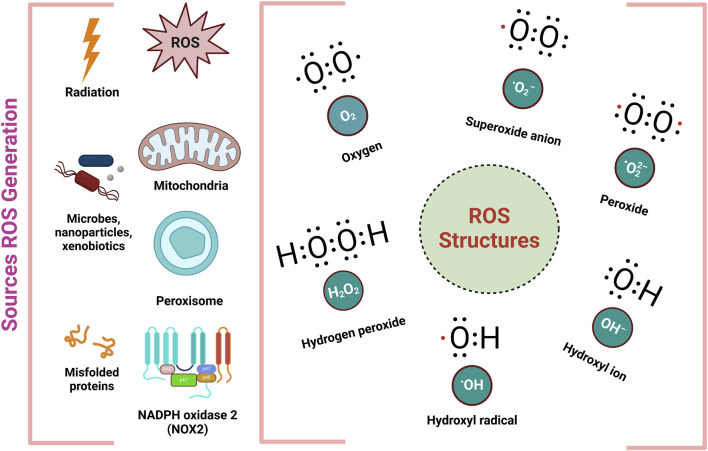
Different sources and structures of ROS (Ahmad et al., 2017).

Additionally the third line of defense focuses on repairing damage and removing harmful substances, facilitating the regeneration of bio-molecules affected by OS (Umber et al., 2023). When ROS and the body antioxidant defenses are out of balance, which leads to oxidative stress, resulting from both external and internal sources. Exogenous sources of pollution include environmental pollution, tobacco smoke, ionizing radiation, household chemicals, and agricultural chemicals like herbicides as well as insecticides (Jomova et al., 2023). Within the body, ROS are generated by mitochondria, cytochrome P450 enzymes linked to the endoplasmic reticulum, membrane-bound NADPH oxidases (NOX 1-5), and peroxisomes (Alwadei, 2023). ROS, including both free and non-free radicals, are mostly produced in regions of elevated oxygen consumption such as mitochondria, peroxisomes, and the endoplasmic reticulum (Figure 3.) (Bouyahya et al., 2024; Santos et al., 2024). This imbalance linked to a number of diseases, such as heart conditions, since OS may harm DNA, lipids, and proteins (Chen S. et al., 2024).

Cardiovascular disorders such as functional hypoxia, metabolic disturbances, uncoupling of mitochondrial electron transport, and inflammation induce oxidative stress in hypertrophied failing hearts. Oxidative stress, induced by these pathogenic stressors, activates metallo-matrix proteases and destroys extracellular matrix proteins, resulting in cardiac remodeling and heart failure. It also causes subcellular remodeling, problems in Ca2^+^ handling, and loss of cardiomyocytes owing to apoptosis, necrosis, and fibrosis. Transient low levels of oxyradical production may stimulate redox-sensitive pathways linked to cardiac hypertrophy, whereas sustained high levels can lead to oxidative stress, calcium handling abnormalities, and protease activation, significantly contributing to detrimental cardiac remodeling, cardiac dysfunction, and the progression of heart failure (Shah et al., 2021).

### 3.1 Mechanism of ROS generation

The mechanism for producing ROS is oxidative phosphorylation, which entails the transfer of electrons from NADH and FADH via four mitochondrial enzymes, culminating in the synthesis of ATP from ADP (Berthiaume et al., 2019). Electrons lost during this process generate superoxide radicals, resulting in functional degradation of tissues and damage to macromolecules. Superoxide and nitric oxide may react to form *peroxynitrite*, a strong oxidant that penetrates membranes and nitrates numerous enzymes. It may also be converted into hydrogen peroxide via superoxide dismutase, generating a highly reactive radical known as hydroxyl radical (Juan et al., 2021;Möller and Denicola, 2024). ROS derived from mitochondria contribute significantly to vascular dysfunction by downregulating *Nrf2*, decreasing NO bioavailability, and increasing the production of vasoconstrictor molecules (Pang et al., 2024). This sequence of events disrupts the mitochondrial membrane, leading to the release pro-apoptotic factors, which results in the apoptosis of plaque cells and inflammation (Chang et al., 2023).

Furthermore, ROS generated by NADPH oxidases during inflammatory reactions are essential for the endogenous production of free radicals (Banerjee et al., 2020). Individuals with a genetic deficit in *NOX2* suffer chronic granulomatous disease, makingthem vulnerable to common infections (Garay et al., 2022). A baseline level of ROS is necessary for maintaining cellular homeostasis; however, excessive ROS can damage cellular macromolecules and are linked to aging and CVDs (Gianazza et al., 2021). Improved endothelial vasodilation following the inhibition of NADPH oxidase with apocynin suggests that endothelial dysfunction is mediated by NADPH oxidation (Leal et al., 2020).

### 3.2 Role of oxidative stress proteins in heart aging

Proteins including albumin, ferritin, ceruloplasmin, and transferrin regulate iron homeostasis, reduce oxidative stress, and preserve endothelial function, all of which are important in heart ageing. These proteins are mostly present in humans and animals. These do not come directly from plants, but they do include substances like iron-binding molecules, antioxidants, and phytochemicals that may affect how these proteins work. Transferrin (Tf), a monomeric glycoprotein that binds iron, is mostly synthesized in the liver. Two moles of iron in two homologous iron-binding domains may bind to 1 mole of transferrin (Talukder, 2021). It is the crucial transport protein that facilitates the movement of iron throughout the bloodstream for most vital processes (Vogt et al., 2021). Tf is internalized by cells via receptor-mediated endocytosis, whereupon it binds to the Tf receptor, functioning as a transporter to maintain intracellular iron homeostasis. Although essential for basic biological processes, iron becomes toxic in excess. Therefore, iron movement into and out of cells is prudently controlled (Leon-Sicairos et al., 2015). A system involving transferrin receptor 1, divalent metal-ion transporter 1, and other proteins carries most of the iron to cells attached to plasma transferrin (Li and Qian, 2002). Transferrin facilitates the controlled distribution of iron to cells by binding and transferring it in the bloodstream. Both an excess and a deficiency of iron may adversely affect cardiac function, making the maintenance of iron equilibrium crucial (Anderson and Vulpe, 2009). Through the Fenton reaction, unbound iron can accelerate the generation of ROS, leading to oxidative damage. Transferrin reduces the availability of free iron for the generation of ROS by sequestering it. The cornerstone of an iron-based mechanism of ischemic preconditioning that protects heart cells from iron-mediated oxidative damage associated with ischemia-reperfusion injury is the synthesis of ferritin, which is prevalent in the H subunit and sequesters redox-active iron (Talukder, 2021). A recent experiment in a mouse cardiomyocyte cell line shown that L-type channels are the principal mediators of iron absorption (Rose et al., 2011).

The importation of ferrous iron via L-type channels, leading to elevated ROS production, may hinder calcium influx, hence affecting cardiac excitation-contraction coupling, which is very sensitive to changes in cellular redox state (Cheng and Lian, 2013). This may therefore lead to suboptimal systolic and diastolic function, symptomatic of iron-overload cardiomyopathy. Recent research shown that iron accumulation mediated by the iron-binding protein Lipocalin-2 leads to cardiomyocyte apoptosis and cardiac remodeling (Xu et al., 2012). Ferritin, play key role in maintaining iron homeostasis and defending cells from iron induced oxidative stress, it is actually a primary iron storage protein (Imam et al., 2017). Ferritin plays key functions in heart aging this includes, iron regulation, cellular senescence, oxidative stress and inflammation (Rosenblum, 2023). Iron deficiency is related to reduced or impaired cardiac function, even deprived of anemia, this leads to reduced exercise capacity, as well as worse consequences in heart failure patients (Jankowska et al., 2010). While iron accumulation such as hemochromatosis, this promotes oxidative stress, fibrosis and cardiomyocyte damage (Mancardi et al., 2021). Iron deficiency is related to reduced or impaired cardiac function, even deprived of anemia, this leads to reduced exercise capacity, as well as worse consequences in heart failure patients (Kremastinos and Farmakis, 2011). While iron accumulation such as hemochromatosis, this promotes oxidative stress, fibrosis and cardiomyocyte damage (Kremastinos and Farmakis, 2011). Through impounding free iron, ferritin supports to mitigate oxidative damage, thus reducing ROS production. Though, when ferritin levels are insufficient or excess, this leads oxidative stress can accelerate heart aging (Alfei et al., 2020).

Ceruloplasmin, plays a key function in heart aging via controlling iron homeostasis and oxidative stress, it is a copper containing ferroxidase (Orzheshkovskyi and Trishchynska, 2019). In heart aging ceruloplasmin helps to mitigate ROS via oxidation of ferrous iron to ferric iron. Therefore, reducing the availability of ferric iron for Fenton reactions, which generate very toxic hydroxy radicals and thus mechanism is key to avoid iron mediated oxidative damage to cardiac tissues (Tian et al., 2022). Additionally, its antioxidant abilities contribute to the upkeep of endothelial function and the decrease of inflammation, both of which are vital for cardiac ageing (Arenas de Larriva et al., 2020). However, dysregulation of ceruloplasmin levels or role may donate to iron overload and heightened oxidative stress, henceforth deteriorating age-related heart dysfunction (Rosenblum, 2023). Numerous studies have shown that the lack of ceruloplasmin is associated with increased cardiovascular risk, underscoring its significance in preserving heart health with ageing (Fox et al., 2000; Vassiliev et al., 2005).

Albumin, plays key roles in heart aging via upholds osmatic pressure, transporting hormones and fatty acids. It is most abundant plasma protein, exerting antioxidant and anti-inflammatory effects (Shastri et al., 2024). Albumin binds and neutralizes free radicals, minimizing oxidative damage to cardiac tissues and lowering oxidative stress and inflammation in the ageing heart (Shastri et al., 2024). The ability of albumin to bind nitric oxide and promote endothelial function is essential for maintaining vascular health and treating age-related conditions including heart failure and atherosclerosis. Albumin, plays key roles in heart aging via upholds osmatic pressure, transporting hormones and fatty acids. It is most abundant plasma protein, exerting antioxidant and anti-inflammatory effects. Albumin binds and neutralizes free radicals, minimizing oxidative damage to cardiac tissues and lowering oxidative stress and inflammation in the ageing heart. The ability of albumin to bind nitric oxide and promote endothelial function is essential for maintaining vascular health and treating age-related conditions including heart failure and atherosclerosis (Belinskaia et al., 2021). Furthermore, its function in balancing and controlling fluid to minimize edema is a crucial factor in cardiac ageing, which is affected (Hankins, 2006). The decreased levels of albumin, often seen in elderly adults, have been linked with an elevated risk for cardiovascular disease, underscoring its protective role in cardiac ageing (Riviati et al., 2024).

## 4 Mitochondrial dysfunction

Mitochondria are double-membraned organelles that contain a circular genome known as mitochondrial DNA (mtDNA) (Ferreira and Rodriguez, 2024). Among the many important tasks they perform is the synthesis of ATP, regulation of nutrient metabolism, calcium homeostasis, and programmed cell death (Perrone et al., 2023). Mitochondria, located in the cytoplasm of eukaryotic cells, function as dynamic networks that perpetually engage in synthesis, fusion, fission, and destruction (mitophagy). Their proper functioning is vital in high energy tissues like the heart, where cardiomyocytes depend on ATP for their activity (Grel et al., 2023). In mature cardiomyocytes, mitochondria produce 95% of the ATP the heart needs and make up over one-third of the intracellular volume (Chang et al., 2023).

Dysfunctional cardiac mitochondria have been identified as a major factor inCVDs, leading to decreased ATP production, increased production of ROS, increased cell apoptosis, and disrupted mitochondrial dynamics (Chistiakov et al., 2018). An essential part of the whole cellular ROS generation process is the activity of mitochondria, originating from the reduction of oxygen (Napolitano et al., 2021; Palma et al., 2024). These ROS can subsequently be converted into H_2_O_2_, which influences the redox state of proteins (Okoye et al., 2023). The generation of mtROS is specific to certain sites and varies depending on the stimuli (Sadiq, 2023). Electrons derived from substrates can reduce O2 to O2⋅− at eight distinct mitochondrial sites, with complex I (CI) and complex III (CIII) being the most significant (Skulachev et al., 2023). Rotenone, an inhibitor of CI, increases O2⋅− production at CI, potentially increasing in mitochondria with lower energy production, elevated ΔpH, higher CoQ ratio, or high NADH-to-NAD^+^ ratio, causing O2⋅− to leak into the mitochondrial matrix (Jiménez-Gómez et al., 2023;Ježek et al., 2024).

Mitochondria possess a complex network of ROS scavenging systems that help regulate OS resulting from mitochondrial ROS (Surai, 2016). These systems include SODs, which convert superoxide radicals into hydrogen peroxide, which is then detoxified by catalase, *GSH-PX*, and the peroxiredoxin/thoredoxin (PRX/Trx) system (Wang et al., 2018; Peoples et al., 2019). SODs serve as the primary defense against mitochondrial ROS, with three isoforms (SOD1, SOD2, and SOD3) managing ROS levels in specific compartments (Eleutherio et al., 2021). Whereas SOD2 is found in the mitochondrial matrix, SOD1 is mostly present in the cytosol and has also been identified in the mitochondrial intermembrane gap (Kim et al., 2017). Proper regulation of the localization and activity of SOD1 and SOD2 is essential for effective mitochondrial ROS scavenging (Mailloux, 2020).

Mitochondrial ROS are essential for cardiac tissue, comprising 45% of the heart’s cellular volume (Kaludercic and Di Lisa, 2020). ROS can activate TFs for instance *NF-kB* as well as *AP-1*, hence facilitating an inflammatory process in tissues (Li A.-L. et al., 2023; Sobhon et al., 2023). Pinpointing the underlying cause of this dysfunction is challenging because of the interconnected nature of various processes (Addo and Khan, 2024). Aging-related mitochondrial dysfunction impacts cells through several simultaneous factors, including elevated mitochondrial ROS production, irregular assembly and recycling of mitochondria, alterations in the quality and quantity of mitochondrial DNA, and changes in the substrates related to mitochondrial respiration (Rai and Fessler, 2024). These molecular indicators are particularly evident in aged cardiovascular cells.

### 4.1 Inflammation

The heart aging process is defined by key features such as progressive hypertrophy of cardiomyocytes, cardiac fibrosis, and inflammation (Hastings et al., 2024). Hypertrophied cardiomyocytes contribute to a hypoxic environment, which produces an abundance of free radicals that may damage cellular constituents (Pena et al., 2020). In response to this stress, cardiomyocytes release pro-inflammatory cytokines and chemokines, initiating an immunological response and augmenting the population of macrophages in the left ventricle (Ciutac and Dawson, 2021; Moskalik et al., 2022). Due to the poor rate of proliferation of mature cardiomyocytes, damaged areas are replaced with fibrotic scar tissue, ultimately leading to organ failure (Bishop et al., 2022). The production of ROS increases with age due to various factors, including physical, chemical, and biological agents, resulting in endothelial dysfunction and cellular damage (Scioli et al., 2020; Pacinella et al., 2022).

Furthermore, when free radicals are produced excessively or uncontrollably, they can trigger an inflammatory response, this is integral to the aging process (Sadiq, 2023). Atherosclerosis, heart problems, diabetes, and other age-related diseases are linked to persistent low-grade inflammation (Candore et al., 2010; Shen et al., 2024). The causes of this syndrome include stress and continuous antigen exposure, leading to a diminished ability to manage stressors and a gradual rise in pro-inflammatory activity (Bennett et al., 2018). The term “inflammaging” refers to the increased inflammatory response that accompanies aging, resulting in a persistent low-grade systemic pro-inflammatory condition (Giunta et al., 2022).

Aging is marked by alterations in the immune, hormonal, and adipose systems, which result in a chronic inflammatory state (Oishi and Manabe, 2016). This chronic inflammatory can lead to issues such as frailty, cognitive decline, and various cardiovascular, neurological, and vascular events (Ijaz et al., 2024). Despite its negative associations, inflammation is crucial for sustaining life and maintaining individual integrity (Ahuja et al., 2023; Baechle et al., 2023). An inflammatory state arises when pro-inflammatory compounds exceed anti-inflammatory controls (Bhol et al., 2024). The origins of this low-grade inflammatory process are still under discussion; however, one theory posits that ongoing stimulation of the immune system drives a pro-inflammatory shift (Gusev and Sarapultsev, 2024). Typically, aging leads toa gradual dysregulation of the immune response, particularly affecting cellular and adaptive immunity, with a notably impact on T cell function (Tobin et al., 2020). The persistent inflammatory condition experienced by elderly individuals is largely caused by this imbalance (Conte et al., 2022). The causes are likely multifactorial, involving chronic stimulation from viruses, bacteria, and endogenous cellular factors, along with ongoing activation of the immune system’s defenses (Endres et al., 2022).

Oral quercetin, administered at 50 mg/kg, significantly mitigates isoproterenol-induced cardiac injury by reducing pro-inflammatory mediators and enhancing anti-inflammatory and antioxidant mediators (Kumar et al., 2017). Quercetin treatment may improve heat stroke outcomes in rats by mitigating hyperthermia and cardiac damage via its anti-lipid peroxidation, antioxidant, and anti-inflammatory effects (Lin et al., 2017). The rat vena cava exhibited comparable outcomes with a reduced dosage but extended treatment duration (20 mg/kg/42 days) via the suppression of reactive oxygen species, the PI3/AKT pathway, and the release of inflammatory cytokines, possibly averting myocardial infarction and ischaemic heart disease. EGCG (40 mg/kg) mitigates myocardial damage in animals by blocking the AKT/mTOR and Hippo pathways (Ma et al., 2019), while enhancing the NQO1 antioxidant pathway (Cui et al., 2021; Huang et al., 2022; Li Y. et al., 2022).

## 5 Mechanism of plant-derived antioxidants on heart aging

The potential therapeutic benefits of plant-derived antioxidants on aging heart and other areas of health have received increasing attention in recent years. This interest arises from the extensive pharmacological properties of several plant compounds, which have been investigated for their ability to slow down aging and its impact on the cardiovascular system. Naturally occurring plant-based products are essential for treating and preventing diseases linked to heart aging (Sreedevi and Mavilavalappil, 2024). For many years, natural plant-based products have been used as active ingredients in conventional medicine (Najmi et al., 2022). Various natural compounds obtained from plants provide a diverse array of biological and pharmacological attributes that are crucial in modern pharmacotherapy (Chaachouay and Zidane, 2024). Among these products are the anticancer medication *paclitaxel*, which is derived from *Taxus brevifolia* (YADAV et al., 2023); the anticancer drugs *vincristine* and *vinblastin*e, which are derived from *catharanthus roseus* (Qu et al., 2019); the anticancer drug *camptothecin*, which is de-rived from *camptotheca acuminata* (Fan et al., 2022); and the anticancer drug quercetin, a polyphenol present in a variety of vegetables and fruits (Rauf et al., 2018).

Plants are believed to require certain nutrients, such as phytochemicals or secondary plant components like polyphenols (Hosoda et al., 2023). These compondsbelong to a large family found in plants and algae, and their primary function is to protect the organism from UV rays, diseases, and herbivore consumption (Del Mondo et al., 2021). Polyphenols possess a variety of structural forms, ranging from simple monomers to intricate polymerized structures (Nagarajan et al., 2020). Seaweed polyphenols have been shown to reduce chronic inflammation (Murray et al., 2021), OS (Begum et al., 2021), hyperglycemia, hyperlipidemia, metabolic abnormalities associated with CVDs, and the aftereffects of diabetes (Gabbia and De Martin, 2020).

Recent research on marine macroalgae has demonstrated that plant-derived polyphenols can improve health outcomes, including reducing the risk of obesity, diabetes, and cardiovascular disease (Pereira and Cotas, 2023). Polyphenols are significant secondary metabolites derivedfrom plants that have notable benefits against cancer, CVDs, diabetes, and neurological disorders (Shahrajabian and Sun, 2023). Various plant species, such as *dipteryx odorata*, *hierochloe odorata*, *galium odoratum*, *dichanthelium clandestinum*, *verbascum* spp., and *anthoxanthum odoratum*, comprise a range of substances, including as tannins, lignins, coumarins, flavonoids, and phenicic acid (Iqbal et al., 2023). Resveratrol, known for its antioxidant properties, has been shown to improve inflammation, cancer, aging, obesity, diabetes, and provide cardio-protective and neurological benefits (Carrizzo et al., 2013; Mohammadi S. et al., 2024). The benefits of antioxidative therapy are increasingly acknowledged as a means of lowering ROS in the vascular and, consequently, decreasing their deleterious consequences (Senoner and Dichtl, 2019).

In addition to their antihypertensive effects, ACE inhibitors decrease circulation Ang II also have detoxifying properties (Dandona et al., 2007). Similarly, statins, or cholesterol-lowering medications, target to control HMG CoA reductase in addition to their cholesterol-lowering effects (Crismaru et al., 2020). To mitigate oxidative damage, vitamins E and C are often used as dietary supplements in addition to other medications (Higgins et al., 2020). Conversely, polyphenols are gaining interest as potential therapeutic agents to lower OS and protect individuals from heart disease (Muscolo et al., 2024). Polyphenols are the most common antioxidant in the diet, consumed ten times more frequently than water-soluble vitamin C and one hundred times more frequently than lipid-soluble vitamin E and carotenoids (Gulcin, 2020). This review study highlights the most extensively studied plant-based antioxidants that have been identified.

### 5.1 Resveratrol

**FIGURE 4:**
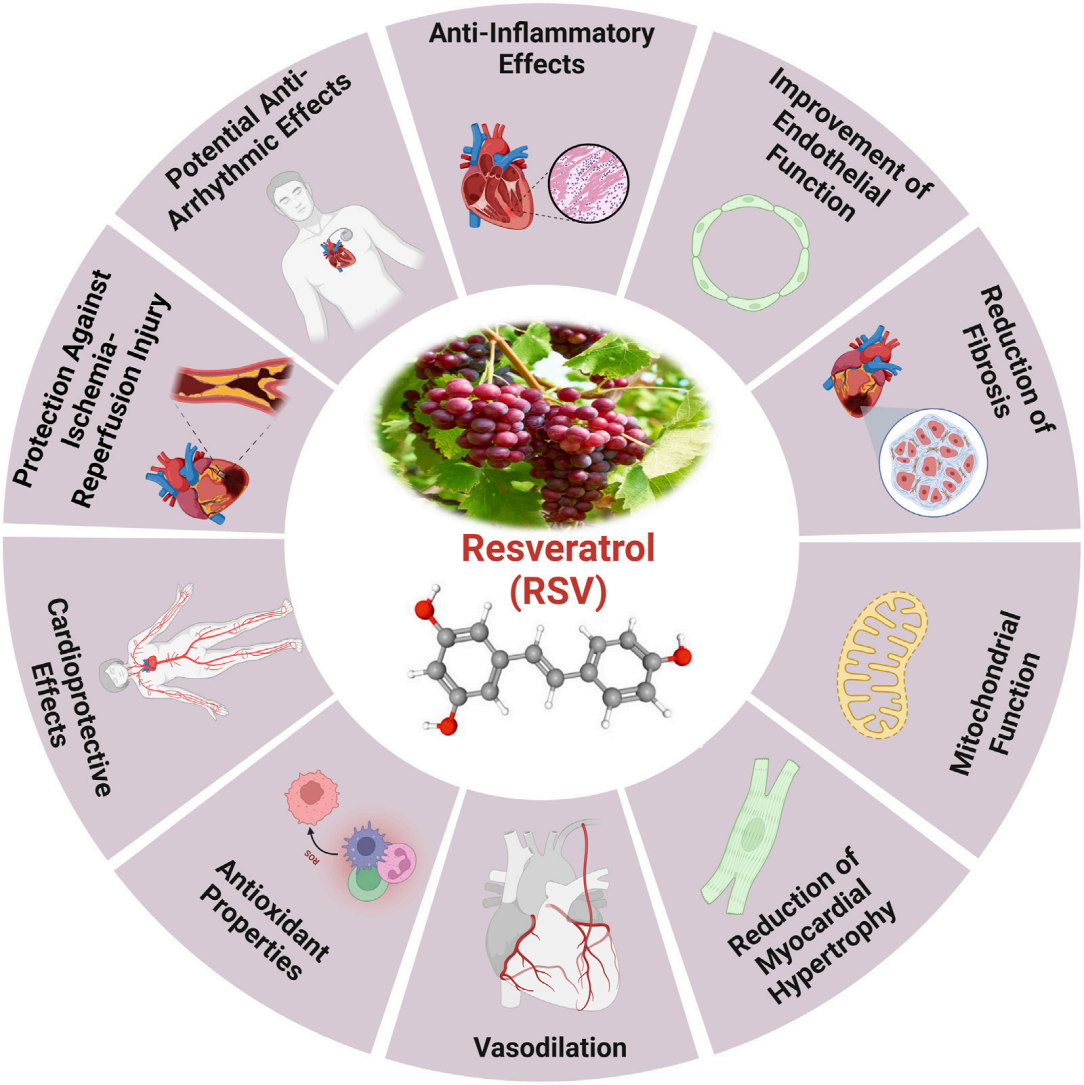
The health benefits of resveratrol and the cardioprotective effects induced by RV (Zhang et al., 2021).

It has been shown that the naturally occurring polyphenol molecule resveratrol, which is present in a variety of plants, may help to maintain cardiovascular health and delay the aging process (Figure 4). A wide variety of plant species, including groundnuts and grapes, contain resveratrol, a naturally occurring stilbene (Kaur et al., 2022). Future studies suggest that resveratrol could serve as a potential chemo-preventive drug due to its ability to inhibit polyphenolic cyclooxygenase. It can be isolated from red wine, grape skins and seeds, and *Polygonum cuspidatum* roots (Kaur et al., 2022). The cardioprotective effects caused by resveratrol are associated with a notable augmentation in antioxidant activity and mitochondrial transmembrane potential, with a decrease in oxidative damage (Yang et al., 2023a).

#### 5.1.1 Mechanistic role of resveratrol

Mechanistically, resveratrol primarily inhibits NADPH oxidase, decreases the production of ROS, and preserves activity of crucial antioxidant enzymes, including SOD, CAT, as well as glutathione peroxidase (Li M. et al., 2024). The modifications induced by resveratrol decrease lipid peroxidation, enhance cardiomyocyte viability, and decrease cardiac hypertrophy (Aires and Delmas, 2015). Resveratrol significantly mitigates cardiac metabolic diseases by the process involves reestablishing glucose homeostasis, regulating free fatty acid oxidation (FFAO), and increasing glucose consumption (Sulaiman et al., 2010). These processes enhance the metabolism of cardiac energy, especially in cardiomyocytes when glucose levels are elevated (Kolwicz Jr and Tian, 2011).

Currently, numerous studies suggest that resveratrol protects mitochondrial oxidation in endothelial cells, supports endothelial function, and it improves blood circulation and cardiac function by promoting vasodilation and vascular angiogenesis (Schmitt et al., 2010). Administration mitigated increased ROS production, MDA levels, the percentage of apoptotic cells, and *Bax* expression, while also enhancing SOD activity in rat CMEC. The substance also demonstrated antioxidative and anti-apoptotic properties by activating *AMPK/Sirt1* (Li J. et al., 2023). The antioxidant properties of resveratrol through *SIRT1* are illustrated by its activation of AMPK (Ungurianu et al., 2023). This activation inhibits NADPH oxidase an enzyme that generates ROS and increases SOD levels, leading to reduced OS (Ilkun and Boudina, 2013). The resveratrol action is attributed to an increase in antioxidant enzyme activity, which varies with age, particularly through modulating crucial pathways (Zhang et al., 2021). Analysis of *SIRT1* revealed that the pathway is silenced in leukocytes treated with resveratrol during aging (Xia et al., 2020). *SIRT1* affects the acetylation of *FOXO* family TFs, which are crucial for lipid and glucose metabolism and cellular response to OS (Nayakanti, 2022). The interaction between *SIRT1* and AMPK enhances *FOXO3* transcriptional activity, thereby increasing *MnSOD* production in cells with higher FOXO3 levels (Ho et al., 2022).

Additionally, resveratrol activated *SIRT3/FOXO3a*-dependent antioxidant enzymes, leading to a reduction in oxidative and DNA damage. Through the activation of the *SIRT3/FOXO3a* signaling pathway, obese mice that were given extended resveratrol demonstrated a decrease in the damage caused by myocardial ischemia (Zhu et al., 2024). By inhibiting NADPH oxidase, ROS production is diminished, thus decreasing OS (Kitamoto et al., 2018). Furthermore, the complex, including *SIRT1* (Hsu et al., 2008), *FOXO3* (Tseng et al., 2013), and *PGC-1α* (Feng et al., 2019), activates *Nrf2* (Kasai et al., 2020), a transcriptional regulator, enhancing antioxidant response gene expression and promoting *MnSOD* production to protect mitochondria from oxidative damage (Zhang et al., 2021). Additionally, resveratrol may effectively inhibit oxidation and inflammation associated with ageing, notably via the antioxidant and anti-inflammatory *Nrf2* pathway (Franco et al., 2025). In aged mice resveratrol reduced the expression of *TLR4*, *NF-κB*, *p65*, and *Notch 1* proteins, leading to lowering pro-inflammatory cytokine levels. This suggesting heart protection depends on suppressing the *Notch/NF-κB* pathway (Sahu et al., 2024). Middle-aged individuals exhibited an improved anti-inflammatory profile in response to resveratrol compared to elderly individuals, particularly in the middle group. This response resulted in a reduction of key biomarkers associated with oxidation and inflammation (Santos et al., 2023).

Furthermore, resveratrol decreased the pH levels in the feces of these mice and increased short-chain fatty acids in the intestinal contents. The production of pro-inflammatory cytokines, specifically *IL-1*β and TNF-κ, was suppressed by injection of resveratrol (Lu et al., 2024). Additionally, resveratrol, melatonin, and their combined use may reverse the reduced antioxidant activity and enhance the expression of *GLUT4*, *SIRT1*, and *PGC-1α* genes in the heart tissue of elderly female diabetic rats. Supplementation with resveratrol and melatonin may help preserve cardiac function in this model of aging female diabetes (Akgun-Unal et al., 2023).

Resveratrol has demonstrated the ability to mitigate age-related ventricular dysfunction by suppressing OS and inflammation in heart tissue via the *Notch/NF-κB* pathway (Sahu et al., 2024). It has been investigated that resveratrol activates the NAD^+-^ dependent protein deacetylase *SIRT1*, reduces the hypertrophy of cardiomyocytes and age-related sarcopenia in mice (Hosoda et al., 2023). Resveratrol therapy resulted in the restoration of autophagic activity in the TA muscle and a reduction in acetylated protein levels (Sin et al., 2016). It may also impede autophagy in the context of cardiac ischemia-reperfusion damage via *DJ-1* regulation of the *MEKK1/JNK* pathway (Liu S. et al., 2023). In addition resveratrol significantly mitigated the loss of *SLC7A11*, inhibited ferroptosis, and improved cardiac function via activating the *Sirt1/p53* pathway in heart failure (Zhang W. et al., 2023).

Furthermore, Resveratrol has been shown to improve lifespan and physical activity in mice with LV pressure, overload-induced hypertension. The investigation elucidates the physiological and molecular processes behind this impact. By decreasing cardiac fibrosis, increasing heart remodeling, and boosting diastolic, vascular, and energy metabolic processes, resveratrol therapy reduces the severity of heart failure in mice (Sung et al., 2015). In addition to maintaining endothelium-dependent coronary artery function and resveratrol improves myocardial perfusion and angiogenesis indicators associated with the VEGF signaling pathway, while also reduces anomalies in wall motion (Robich et al., 2010). In the context of cardiac ischemia/reperfusion damage, the investigation evaluated the impact of resveratrol on *STIM1*-mediated intracellular Ca^2+^ buildup and cell death. Resveratrol dramatically enhanced heart function, lowered infarct size, and decreased apoptosis in mice. Resveratrol reduced intracellular Ca^2+^ buildup and downregulated *STIM1* expression in rat ventricular cardiomyocytes. *In vitro*, *STIM1* over-expression enhanced the effects of resveratrol on *STIM1*-mediated intracellular Ca^2+^ buildup, while the *SOCE* inhibitor *SKF96365* partially eliminated these effects (Xu et al., 2019).

In another study it has investigated that resveratrol play a key role in modulating ferroptosis and cardiac damage in MI. Resveratrol reduced myocardial damage and fibrosis associated with MI in rats, inhibited *IL-6*, *IL-1*β levels, decreased *GPX4* and *SLC7A11* expression. It alleviated cardiomyocyte damage generated by oxygen-glucose deprivation and inhibited ferroptosis in cardiomyocytes under OGD conditions *in vitro*. Resveratrol mitigated myocardial damage by suppressing ferroptosis via the activation of *KAT5/GPX4* in MI, offering additional evidence for its potential therapeutic efficacy (Liu J. et al., 2022). Furthermore, the administration of resveratrol protected cells from *LPS*-induced apoptotic cell death by reducing proinflammatory cytokine generation, increasing *Nrf2* activation in human heart cells, and mitigating *LPS*-induced heart damage in rats (Kosuru et al., 2018a).

Extended resveratrol consumption may protect obese mice from myocardial ischemia damage by restoring intracellular redox equilibrium through the activation of the *SIRT3/FOXO3a* signaling pathway (Zhu et al., 2024). Resveratrol cardioprotective effects in older mice include increased antioxidant activity, mitochondrial transmembrane potential, and decreased oxidative damage. It inhibits pro-inflammatory cytokines and suppresses the *Notch/NF-κB* pathway, enhancing its cardioprotective properties (Bohara et al., 2022; Peng et al., 2023).

It has been established that resveratrol activates *Sirt1*, which in turn mediates the deacetylation of *Smad3* and suppresses the fibrotic response generated by *TGF-β1* (Chen et al., 2015). The level of acetylation of *Smad3* (*Ac-Smad3*) was elevated in rats with cardiac fibrosis, and renal fibrosis but it was reduced in the normal myocardium and nephridial tissue of rats. The Ac-Smad3 has the ability to control the DNA binding activity and transcriptional activity of certain profibrotic genes. Elevating the level of *Ac-Smad3* through the action of *TGF-β1* facilitates the progression and advancement of tissue fibrosis (Chen et al., 2015; Chen Q. et al., 2022). Resveratrol has demonstrated not only its ability to slow down the aging process, but also its ability to provide protection against CVD by eliminating ROS and improving the functioning of several antioxidant enzymes.

### 5.2 Curcumin

**FIGURE 5:**
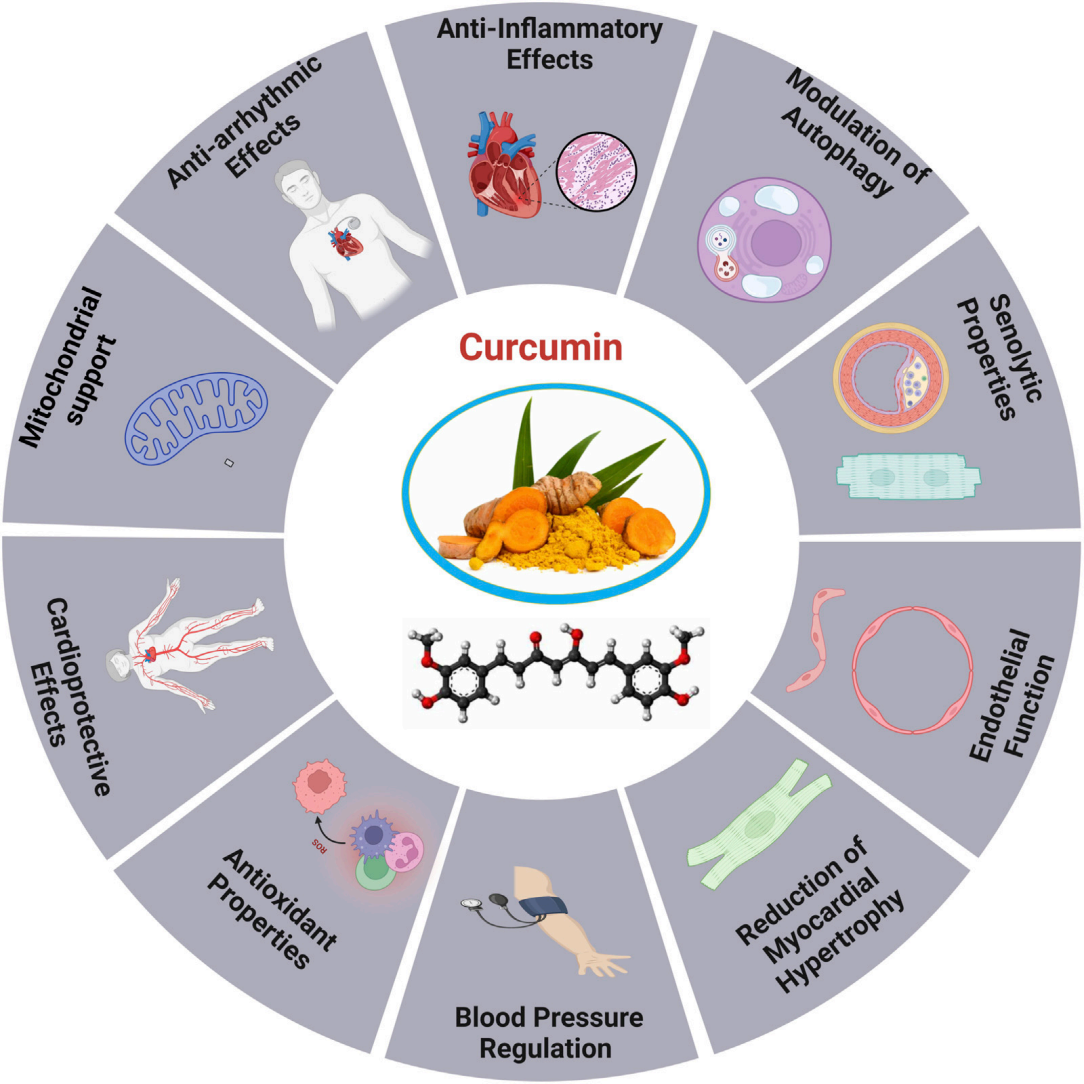
Cardioprotective roles of curcumin (Srivastava and Mehta, 2009).

Curcumin, a naturally occurring substance obtained from the desiccated rhizomes of *Curcuma longa* L., commonly known as turmeric, is extensively used in medical practice to address an extensive range of ailments (Figure 5). Several studies have demonstrated that curcumin has positive effects on cardiac conditions and endothelial system dysfunction (Cox et al., 2022; Ghorbanzadeh et al., 2022). Research on a rat model of hypertension and ischemia have shown that curcumin may improve cardiac hemodynamic function and attenuate heart failure (Aziz et al., 2022). Additionally, by reducing OS and inflammation, it increases myocardial infarction size and boosts cardiac function after ischemia events (Hori and Nishida, 2009).

#### 5.2.1 Mechanistic role of curcumin

Curcumin has garnered interest in the realm of CVDs for its ability to provide variety of beneficial effects on the heart. Due to its antioxidant properties, curcumin has been proposed to mitigate heart disease risk factors, such as cardiac angiogenesis, and delay the development of age-related disorders (Rysz et al., 2021; Aziz et al., 2022). Curcumin, administered at 100 mg/kg, increased O-GlcNAcylation and the production of O-linked N-acetylglucosamine transferase in *HaCaT* cells, therefore improving the stability of apolipoprotein C3, a substrate of O-linked N-acetylglucosamine transferase (Sun et al., 2025). For instance, curcumin contributes to heart aging by promoting autophagy and restoring it through the *SIRT1/AMPK/mTOR* pathway. The antioxidant capacity of *SIRT1* was reduced by siRNA-mediated knockdown, highlighting its anti-aging, and autophagy-enhancing effects, indicating its potential as an effective treatment for cardiac aging (Yang et al., 2022). The study explores the involvement of the *Nrf2/HO-1* signaling pathway in DCM by employing curcumin and *shRNA-Nrf2* as activators. A Type 2 diabetes animal model induced by streptozotocin and a high-fat diet was used to assess the impact of curcumin on *H9C2* cells. The results indicated that excessive production of ROS impaired *Nrf2*-related signaling, leading to reduced cellular energy metabolism and increased apoptosis. In contrast, activating the *Nrf2* signaling pathway improved cardiomyocyte viability, decreased ROS generation, and inhibited apoptosis (Wu et al., 2022).

Moreover, Curcumin enhances mitochondrial integrity, mitigates oxidative stress, and promotes mitochondrial biogenesis by activating *SIRT1* and *PGC-1α*. It also prevents the translocation of *DRP1* in sepsis models, indicating potential therapeutic advantages for sepsis-related cardiac muscle (SCM). The *SIRT1-DRP1/PGC-1α* pathway involved in regulating mitochondrial mass may constitute a prospective target for the advancement of organ-protective pharmacotherapies in critical care environments (Hou et al., 2024).

Additionally, curcumin, a natural remedy, has been shown to increase the longevity of postmitotic cells even in the absence of mitochondria, although it does not exhibit hormetic effects. Its mechanism includes the inhibition of *TORC1* activity, elevated ATP levels and the onset of oxidative damage, indicating potential therapeutic uses in age-related diseases (Naaz et al., 2024). Curcumin supplementation can reduce vascular OS and restore arterial function in aging, positioning it as a promising antioxidant therapy for addressing age-related arterial dysfunction (Fleenor et al., 2013). The treatment of curcumin increased *VEGF-A*, *TSP-1*, and *NF-κB* levels and boosted age-related decreases in angiogenesis. By upregulating the production of *VEGF* and *NF-κB* proteins and downregulating *TSP-1* protein levels, it mitigates heart tissue damage and supports cardiac angiogenesis in diabetic rats. Curcumin also induced molecular changes lead to a reduced apoptosis index in cardiac tissue (Ghorbanzadeh et al., 2022). The study revealed a substantial drop in autophagy and *SIRT-1* levels, while the levels of *MDA*, *NOX4*, *p-NF-κb*, and *P62* were considerably elevated in the heart of the old group compared to the young group (Aziz et al., 2022; Lv et al., 2022).

Additionally, it was found that the hearts of older rats exhibited notably elevated levels of apoptosis and fibrosis in comparison to younger rats. However, the administration of exercise and curcumin shown a positive effect in ameliorating these negative alterations. The combined treatment of curcumin and exercise in elderly rats had a more pronounced impact on molecular mediators and histological alterations in the heart than the use of curcumin alone (Dong et al., 2020).

Through its capacity to activate and repair autophagy curcumin influences heart aging through the *SIRT1/AMPK/mTOR* mechanism. In aged cardiomyocytes subjected to D-galactose treatment, there was a significant increase in the number of cells that tested positive for intracellular ROS, P53, P16, and senescence-associated β-galactosidase. Curcumin-induced autophagy elevated SIRT1 and AMPK levels, while reducing *mTOR*. *SIRT1-siRNA* stimulated the *SIRT1/AMPK/mTOR* pathway, limiting the antioxidative, antiaging, and autophagy-enhancing effects of curcumin in a dose-dependent manner (Yang et al., 2022). Thymoquinone and curcumin synergistically reduced *D-gal* induced necrosis in the brain and heart, leading to a reduction in *caspase-3*, *calbindin*, *IBA1*, cardiac *caspase-3*, and *BCL2* levels. The combination reduced mRNA expression of *TP53*, *p21, Bax, and CASP-3* in the brain and heart, while enhancing *BCL2* expression relative to the *D-gal* group. This indicates that *TQ* and curcumin provide a viable approach for mitigating aging (Dong et al., 2020).

### 5.3 Quercetin

**FIGURE 6:**
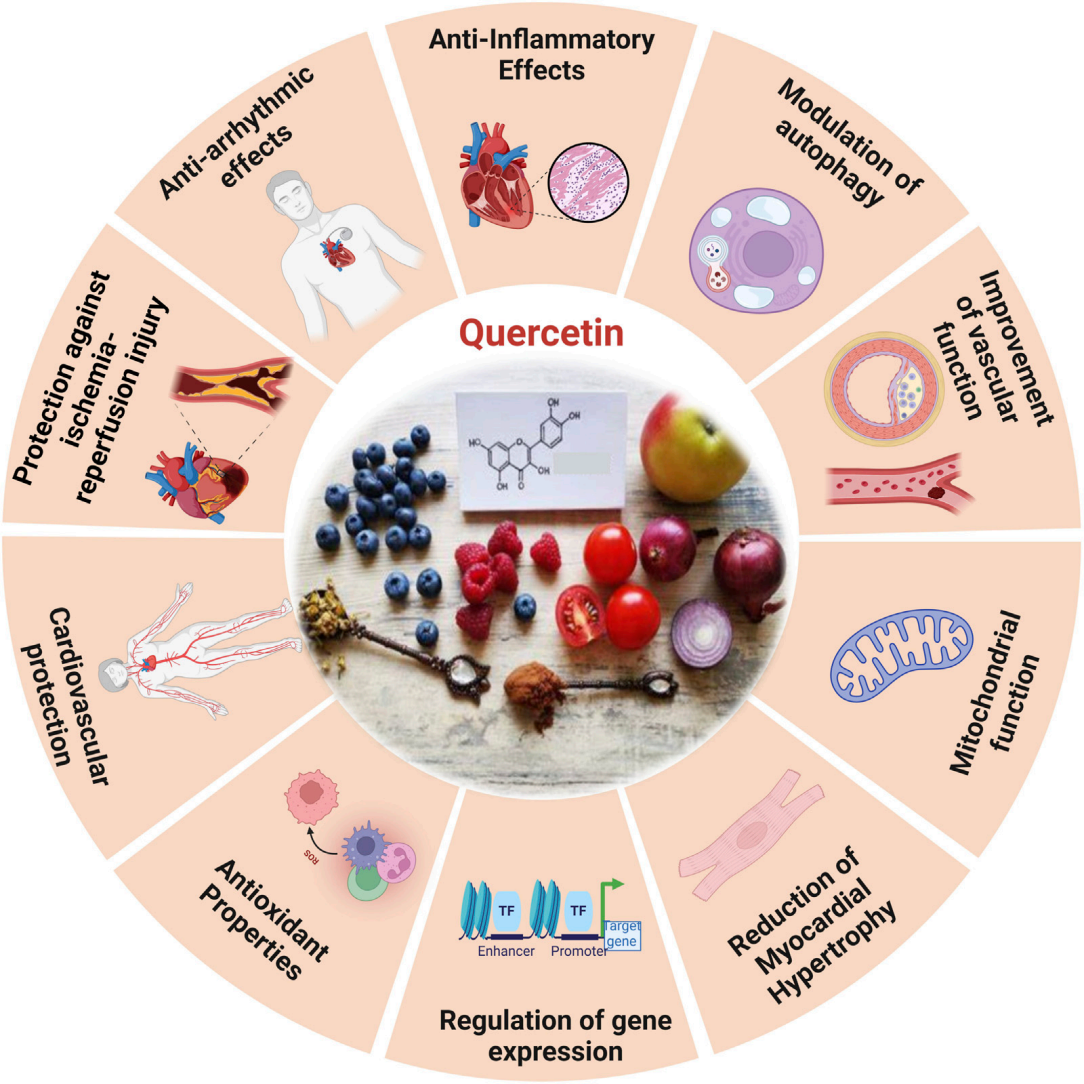
The diverse functional role of quercetin and the cardioprotective effects induced by quercetin (Ferenczyova et al., 2020).

One of the most well-known dietary antioxidants is quercetin, a phenolic member of the flavonoid family that is crucial to the process of heart aging (Figure 6). It is found in vegetables, fruits, tea, wine, and many other healthy goods (Carrillo-Martinez et al., 2024). The antioxidant effects of quercetin include scavenging free radicals such superoxide, hydrogen peroxide, peroxyl, and hydroxyl (Cizmarova et al., 2023).

#### 5.3.1 Mechanism of quercetin

Quercetin, a non-toxic flavonoid with antioxidant, anti-apoptotic, and anti-inflammatory characteristics, is essential in the treatment of aging-related disorders within a safe dosage range. Quercetin improves mice left ventricle function (Ulasova et al., 2013), myofibrillar tissue, and mitochondrial structure by lowering OS brought on by aging, restoring myocardial microcirculation, and decreasing the size of infarcts (Gao et al., 2014). The study reveals that in rats with myocardial infarction, quercetin increases the activity of enzymes related to the respiratory chain and the tricarboxylic acid cycle (Cui et al., 2022). The inhibitory impact of quercetin on cellular senescence is associated with the preservation of MERCs and enhanced mitochondrial activity, which may mitigate cardiac failure (Jiménez et al., 2025). The production of biomarkers linked to OS generated by myocardial infarction in rats has been observed to decrease (Albadrani et al., 2021).

Moreover, quercetin has been shown to defend *AC16* cells against OS caused by hyperlipidemia in elevating *p-SIRT1* levels, enhancing endothelial *NOS*, and diminishing *iNOS* (Cui et al., 2022). This defensive action is facilitated by the signaling pathway *PI3K/Akt/Nrf2* (Srivastava et al., 2023), by obstructing the *HMGB1-TLR4-NF-κB* signaling pathway, quercetin efficiently reduces inflammatory responses (Wang et al., 2021).

Studies indicated that quercetin markedly moderates cardiac hypertrophy and fibrosis in spontaneously hypertensive rats, this material preserves mitochondrial integrity, lowers levels of mitochondrial superoxide, and enhances heart performance.

In addition, *in vitro* studies demonstrated quercetin alleviated hypertrophic response in rats by maintaining mitochondrial function, while partially weakened mitochondrial protection and *PARP-1* inhibition after *SIRT3* knockdown. Quercetin reduces cardiac hypertrophy by increasing mitochondrial activity by regulating the *SIRT3/PARP-1* pathway, according to study (Chen et al., 2021). Prolonged treatment with quercetin in older spontaneously hypertensive rats (SHRs) markedly inhibited *MYC* expression, reduced *CYP2E1* levels, and decreased lipid peroxidation. Quercetin amplifies antioxidant activity, improving the equilibrium between prooxidants and antioxidants in the heart, which may result in reduced blood pressure and relative heart weight in older spontaneously hypertensive rats administered quercetin (Maksymchuk et al., 2023). Quercetin decreased MMP activity, *TGF-β* levels, and OS in the coronary arteries and left ventricles of *2K1C* rats. However, it had no effect on hypertrophic remodeling or functioning in the coronary arteries damaged by hypertension (Da Rocha et al., 2023). Quercetin pretreatment activates the *PI3K/AKT* signaling pathway, alleviates CDDP-induced oxidative stress, protects mitochondrial function, and lowers mitochondrial apoptosis in PCs. *In vitro* BLB models show quercetin diminishes CDDP-induced apoptosis and improves endothelial barrier permeability (Huang et al., 2024).

Quercetin reduces apoptosis *in vivo* by phosphorylating *JNK* and *p38*, upregulating *Bcl-2* expression, and inhibits the activation of *Bax* and *caspase-3* (Jubaidi et al., 2021), and via *SIRT1/PGC-1α* signaling, quercetin inhibits MI/R-induced apoptosis (Cui et al., 2022). Atherosclerosis is primarily caused by endothelial dysfunction, which occurs during the solute exchange between blood and nerve tissues. The peripheral nerve substructures are defended by the blood-nerve barrier (BNB), which is made up of endothelium. Atherosclerosis is exacerbated by oxidative injury to endothelial cells induced by oxidized low-density lipo-protein. Consequently, oxLDL promotes the development of foam cells derived from *RAW264.7* macrophages, which worsen cellular lipid accumulation and increase ROS levels that result in the oxidation of LDL particles into ox-LDL. Quercetin could inhibit the production of foam cells generated by ox-LDL and prevent cellular senescence (Cao et al., 2019). In contrast, quercetin hinders the apoptosis of macrophages induced by cholesterol accumulation, thus resulting in a reduction in atherosclerosis. Quercetin additionally enhances the antioxidant function of cells via the Nrf2 pathway (Lu et al., 2017).

Moreover, chronic atherosclerosis throughout aging stimulates the formation and buildup of ROS, leading to mitochondrial damage caused by damage to mitochondrial DNA (mtDNA) (Shemiakova et al., 2020). OxLDL molecules are connected to *NF-κB*, *TLR*, and scavenger receptors, among other pattern recognition receptors, and have the capacity to activate the immune system (Bhaskar et al., 2016). Quercetin, on the other hand, effectively prevents the ox-LDL-containing macrophages from activating *NLRP3* inflammatory vesicles leading to a reduction in cell lipoatrophy and the secretion of *IL-1*β (Cui et al., 2022; Xu et al., 2024). It markedly decreased *VCAM-1* and *ICAM-1* expression in *HUVECs*, downregulated *MCP-1* mRNA levels, and mitigated nuclear translocation of the *NF-κB*, *p65* subunit in oxLDL-stimulated *HUVECs*. Additionally, quercetin reduced *TLR2* and *TLR4* expression, diminished inflammatory mediators, and mitigated the inflammatory process in atherosclerotic rats subjected to a hypercholesterolemic diet. Quercetin functions as an anti-inflammatory and anti-atherogenic compound (Bhaskar et al., 2016). The primary mechanism by which quercetin inhibits the development of atherosclerotic plaque is by controlling *caspase-3* and *NF-κB* activation through the *PI3K/AKT* pathway (Lu et al., 2017).

### 5.4 Epigallocatechin gallate (EGCG)

**FIGURE 7:**
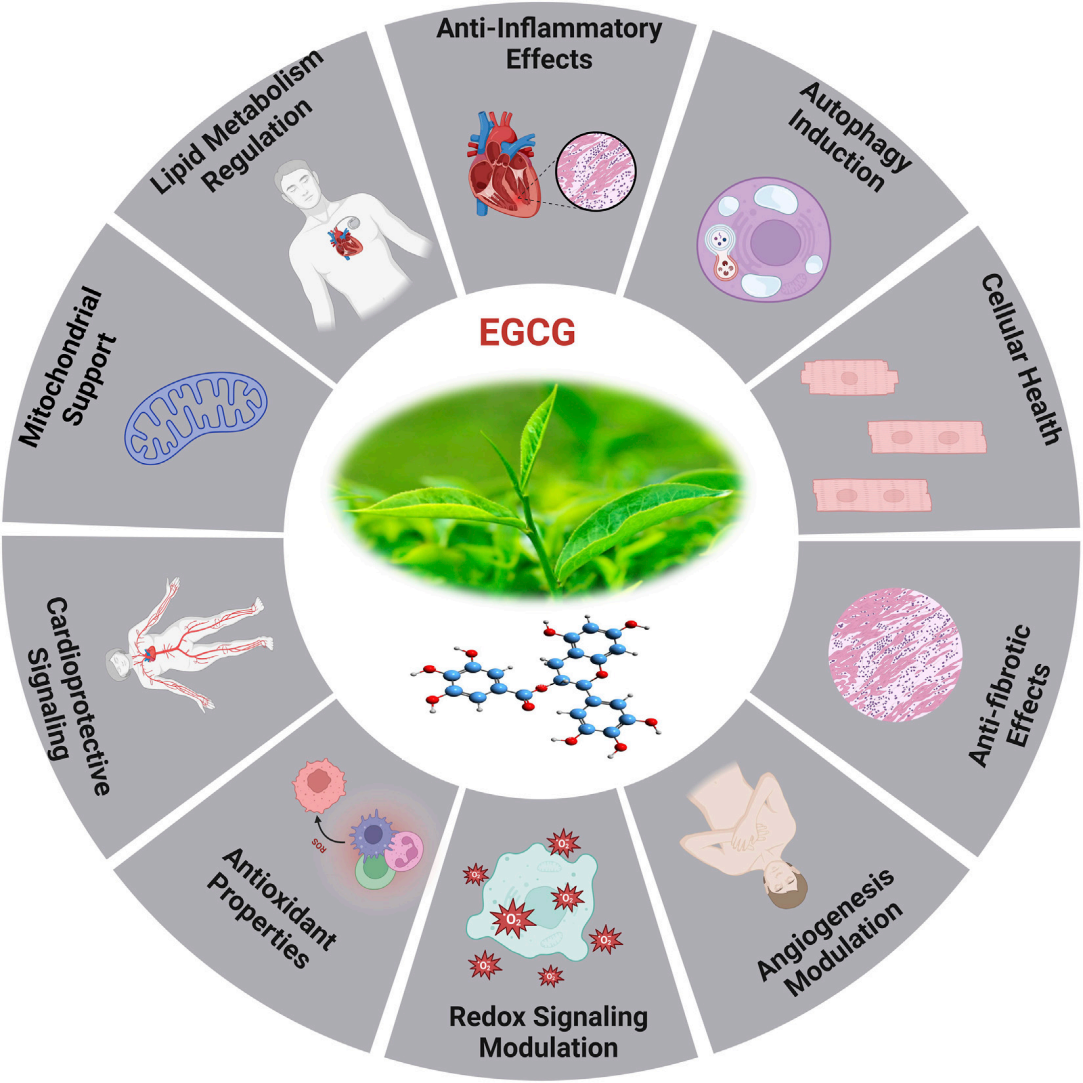
The diverse efficient roles of EGCG and the cardioprotective effects induced by EGCG (Zheng et al., 2011).

Epigallocatechin gallate (EGCG) is the predominant and physiologically active polyphenol found in green tea (Eng et al., 2018). As a strong redox agent, EGCG has a strong antioxidant effect and plays a significant role in heart aging (Figure 7). Its structural phenolic hydroxyl group oxidizes to produce a relatively stable molecule and serves as a hydrogen source for redox reactions. This procedure successfully rids the body of a significant amount of harmful free radicals (Singh et al., 2011).

#### 5.4.1 Mechanism of EGCG

Mechanistic investigations through its modification of MAPK, *PKC*, and *PI3K* activity, EGCG protects cells against oxidative damage, according to mechanistic studies (Zhao et al., 2018). EGCG possesses anti-oxidant properties and can mitigate the inflammatory response through its impact on inflammation-related pathways, resulting in suppression of inflammatory factor expression (Mokra et al., 2022). The research indicates that EGCG could reduce vascular calcification via influencing the MAPK-JunB pathway, with the downregulation of JunB being essential. Ingesting green tea or EGCG medicine may elevate EGCG concentrations, perhaps addressing JunB for the prevention and therapy of vascular calcification (Li et al., 2025). *In vitro* studies show that EGCG decreases inflammatory factors caused by LPS via phosphorylating signaling molecules associated to the *NF-κB/p65*, *MAPK/p38*, Akt, and ERK pathways, as well as by reducing the production of *iNOS* and *COX-2* (Zhong et al., 2012; Kim et al., 2022). Furthermore, EGCG has the potential to decelerate the aging process through its modulation of the AMPK pathway, mitochondrial function restoration, and induction of autophagy (Xu et al., 2023).

Oxidative stress caused by pressure overload-induced cardiac hypertrophy shortens telomeres in the hypertrophic myocardium and depletes *TRF2*. EGCG, a powerful antioxidant, may impede cardiac myocyte death by averting telomere shortening and the loss of *TRF2* (Eng et al., 2018; Brandt et al., 2022). An investigation has revealed that aged rodents treated for 8 weeks with EGCG exhibited enhanced cardiac diastolic function (Oyama et al., 2017). In the aged myocardium, EGCG therapy restored the reduced expression of *cTnI*, and lowered *HDAC1* and *HDAC3* expression and *HDAC1* binding in the proximal promoter of *cTnI*. Additionally, higher concentrations of *AcH3K9* were found in the *cTnI* promoter, following EGCG treatment. In response to EGCG, transcription factors *GATA4* and *Mef2c* bound to the *cTnI* promoter at higher amounts (Pan et al., 2017). The *cTnI* gene plays a crucial role in regulating heart function, particularly in relation to diastolic function (Pan et al., 2016), and deficiencies and mutations in *cTnI* have been associated with diastolic dysfunction and HEpEF (Heinzel et al., 2020). The examination of limited samples of human heart tissues reveals a reduction in *cTnI* concentration in left ventricular myocardial cells in elderly adults, irrespective of cardiac disease status (Myhre et al., 2019). It has revealed that a decrease in cTnI in aging hearts may be a contributing factor to the diastolic dysfunction observed in elderly mice.

The EGCG not only affects the sensitivity of myofilament Ca2^+^, but it also controls gene ex-pression through epigenetic alterations. The study demonstrated that the administration of EGCG reversed the decrease in *cTnI* expression, which is linked to age-related cardiac diastolic dysfunction. This was achieved by increasing the expression of acetylated lysine-9 on histone H3 in aging hearts. The additional it has suggested that the administration of EGCG may have the potential to prevent heart failure through the modulation of histone acetylation (Quan et al., 2024). EGCG exhibits antioxidant activity in *HepG2* cells and offers protection against oxidative stress caused by ABAP. In a dose-dependent way, the procedure was accomplished by lowering ROS and increasing the activity of cellular antioxidant enzymes such as SOD, CAT, and *GSH-Px*. Theoretical foundations for the creation of functional food components can be derived from the antioxidant effects exhibited by combinations of EGCG (Zhang Q. et al., 2023). The therapeutic impacts of EGCG on CVD are associated with its capability to reduce LDL cholesterol, *NF-κB*, plasma glucose, glycated hemoglobin levels, myeloperoxidase activity, inflammatory indicators, and ROS formation. For example, the combination of EGCG consumption and regular exercise in postmenopausal women who are over-weight or obese resulted in a decrease in their resting heart rate (Eng et al., 2018). A study involving randomized controlled trials found that green tea consumption or low polyphenol dosage significantly reduced systolic and diastolic blood pressure in humans by 1.98 and 1.92 mmHg, respectively. The findings of a randomized double-blind placebo-controlled cross-over study demonstrated that the administration of a single dose of 300 mg EGCG resulted in the amelioration of endothelial function and enhancement of arterial-mediated dilation in individuals diagnosed with coronary arterial diseases. However, the administration of 150 mg of EGCG twice daily for a duration of 2 weeks did not yield any statistically significant effects (Mahdavi-Roshan et al., 2020).

Recently investigated study on the impact of *HDAC1*-mediated deacetylation of *NRF1* on cardiac hypertrophy and mitochondrial stability. An *HDAC1* inhibitor called EGCG was reported to enhance *LysoTracker + cardiomyocytes* in hypertrophic circumstances, decrease heart-to-body weight ratios, and improve cardiac function. In hypertrophic *H9C2* cells treated with PE, EGCG decreases cell hypertrophy and increases the presence of *LC3B II + MitoTracker + puncta*. It also inhibits *HDAC1*-mediated histone deacetylation, which aids in maintaining NRF1 levels (Li G. et al., 2024). Eight weeks of EGCG treatment significantly reduced systolic, diastolic, and mean arterial pressure while increasing the liasodilator-hypertensive ratio. This suggests a shift towards sympathetic nervous system dominance or reduced parasympathetic nervous system activity. The results may be attributed to EGCG acting as a sympathetic potentiator or compensatory response. SBP was linked to obesity and insulin resistance, while DBP showed a positive association with HF nu and a negative correlation with LF ms2, this highlighted EGCG’s potential protective effects against hypertension (Wilasrusmee et al., 2024).

In addition, study on adipose-derived stem cells (ADSC) pretreated with EGCG enhanced cell functions in diabetic cardiomyopathy while inhibiting the effects of small interfering C-X-C motif chemokine receptor 4 (siCXCR4) administration. These results were validated in a diabetic animal model, indicating that EGCG-pretreated ADSC may have promising clinical applications to diabetic patients suffering from cardiomyopathy (Chen T.-S. et al., 2024). EGCG is crucial for epigenetic regulation and can reduce DNA hypomethylation in genes like *Sod2*, *Gpx1*, *Cat*, and *TrxR*. The antioxidant properties and epigenetic modifications in CpG methylation can aid in the administration of antioxidant substances and DNA methylation-modifying medicines for chronic disease prevention and treatment (Banzubaze et al., 2024).

## 6 Other plant-derived antioxidants

The impact of plant-derived antioxidants on heart aging has garnered significant attention in scientific research. This interest is driven by mounting evidence showing the potential benefits of several plant-based components, including flavonoids, polyphenols, and other phytochemicals, in relation to the changes in the heart caused by aging. These substances exhibit a variety of mechanisms that are essential for maintaining cardiovascular function. For instance, many plant-derived compounds demonstrate antioxidant properties that can effectively counteract oxidative stress, a critical factor in the senescence of the cardiovascular system (Corrêa et al., 2018; Akbari et al., 2022).

### 6.1 Anthocyanins

Anthocyanins, found in vibrant fruits and vegetables, have recently been the subject of studies exploring their potential, and mechanisms in human vascular endothelial cells and rat thoracic aortas subjected to aging models (Wallace, 2011). These studies examined the ability of specific anthocyanins, such as cyanidin-3-rutinoside (C-3-R) and cyanidin-3-glucoside (C-3-G), to inhibit senescence induced by d-galactose in human endothelial cells (Lee et al., 2020). The findings demonstrated a reduction in the activity of certain markers of cellular senescence, as well as a suppression of ROS production and NADPH oxidase activity in the presence of D-galactose (Hafez et al., 2024). Anthocyanins were shown to counteract the inhibition of endothelial *eNOS* activity, leading to the recovery of NO levels in endothelial cells (Edirisinghe et al., 2011). It was observed that anthocyanins induced *eNOS* deacetylation via *SIRT1*, resulting in increased *eNOS* activity. In an *in vivo* study involving aged rats, administration of anthocyanin-rich mulberry extract over 8 weeks led to a reduction in oxidative stress and endothelial senescence in the aorta, as well as an increase in serum NO levels, *eNOS* phosphorylation, *SIRT1* expression, alongside a decrease in nitro-tyrosine levels in the aorta (Lee et al., 2020).

Additionally consuming anthocyanins has been shown to enhance antioxidant defense enzymes, total antioxidant capacity, and the antioxidant properties of HDL in both preclinical and clinical populations (Garcia and Blesso, 2021). Anthocyanins exhibit direct antioxidant capabilities and indirectly stimulate *Nrf2* and antioxidant gene expression, mitigating oxidative stress and inflammatory signaling in atherosclerotic plaque cells, such as macrophages and endothelial cells. This may potentially protecting against atherosclerosis and cardiovascular disease (Mohammadi N. et al., 2024).

### 6.2 Allicin

Scientifically known as s-allyl-2-ene-1-thiosulfite, allicin is a physiologically active compound derived garlic. It demonstrates a broad spectrum of pharmacological characteristics, such as immunoregulatory, antioxidant, anti-inflammatory, renal, nerve, and cardiac protective, as well as anti-tumor effects. It has been shown that allicin and its derivatives operate biologically by modifying gene expression and interacting with a variety of signaling pathways (El-Saber Batiha et al., 2020; Gao et al., 2024). Allicin, a medication for myocardial infarction, reduces infarction area and fibrosis, increases *SHP2* protein levels, and inhibits ROS in infarction tissue. However, specific knockdown of *SHP2* negates ROS changes. Allicin also modulates *p-PERK* activation, mitigate oxidative stress in rodents (Gao et al., 2024). The study revealed that allicin exerted effects on cardiac function, myocardial fibrosis, and the modulation of *NF-κB* signaling pathways in the myocardial tissue of rats afflicted with diabetic cardiomyopathy. The study provided evidence that allicin exhibited positive effects on cardiac dysfunction and reduced myocardial fibrosis in the rats, possibly via facilitating the deactivation of the *NF-κB* signaling cascade (Ma et al., 2017).

### 6.3 Ginkgolides biloba

The longevity of *G. biloba L*. has led to its widespread recognition as a living fossil tree. Throughout its lifespan, *G. biloba L*. is presumed to have acquired or evolved resistance to many diseases as a mechanism of adjusting to its surroundings. Many different phytochemicals, such as flavonoids, terpenoids, alkylphenols, and carboxylic acids, are present in the leaves of *G. biloba* (van Beek and Montoro, 2009; Liu X.-G. et al., 2022). Only G. biloba trees contain ginkgolides A, B, C, and J. Ginkgo contains mono-, di-, and tri-glycosides as its main flavonoids (Medicine et al., 2021). Ginkgo leaf contains several chemical compounds that have different functions, such as removing oxygen free radicals and decreasing oxidation, regulating superoxide dismutase and catalases, and removing NO. Engaging in these activities has the potential to enhance protection against heart injury and potentially reduce the likelihood of MI (Kadhim et al., 2020).

Moreover, *G. biloba* has the ability to stimulate the *AKT* signaling pathway, the activation of AKT initiates cell-specific processes, such as *GSK3β* phosphorylation, which protect cells from acute AMI damage and reduces the *AKT* signaling pathway due to cardiac ischemia-reperfusion damage (Cho et al., 2009; Lejri et al., 2019). The GBE50 is an orally given GBE formulation that corresponds to the German product, EGb761, which has been used in the treatment of AMI (Liu et al., 2013). While GBE80 activates the *AKT/GSK3β/β*-catenin signaling pathway, successfully preventing myocardial damage from AMI and H_2_O_2_-generated cardiomyocyte cytotoxicity (Zheng et al., 2021).

Additionally, the effects of GBE administration on autophagy and cardiac hypertrophy may be mitigated by the *SIRT1* inhibitor *EX-527*, which also lowers Ang II oxidative stress and the production of *SIRT1* and *FoxO1*. This implies that GBE may be useful as a medication to treat pathological heart hypertrophy (Jiang et al., 2021). GBE has demonstrated cardioprotective properties in individuals with diabetes, namely, in the context of DCM. Investigation demonstrates that giving diabetic rats GBE treatment successfully lowers metabolic irregularities, enhances cardiac function, and lessens degenerative changes to the heart. GBE treatment, however, may be able to address defective autophagy and dysregulation of the *AMPK/mTOR* signaling pathway. GBE demonstrated a reduction in apoptosis produced by *HG* in *H9C2* cells *in vitro* (Yang et al., 2023b). The study establishes a correlation between heightened cardiac oxidative stress, inflammation, apoptosis, and histo-morphological alterations in cardiotoxicity generated by *Cs-A*.

Furthermore, GBE administration has been shown to reduce the cardiotoxic effects of *Cs-A* by activating the *mTOR/ERK1/2* signaling pathways. The aforementioned pathways are linked to the suppression of oxidative stress and inflammatory mechanisms, thereby serving as a preventive measure against heart injury. Immunohistochemistry and bio-chemical assays were employed in the study to demonstrate that the supplementation of GBE leads to a reduction in cardiotoxicity through the enhancement of the *mTOR/ERK1/2* signaling pathways (Asiwe et al., 2024). The extract of GBE shown significant efficacy in mitigating myocardial infarction through the enhancement of the body’s inherent antioxidant defense mechanism and the reduction of inflammatory cytokine release and heart injury marker enzymes. The leaves of GBE, specifically *EGb761*, are commonly used to treat cerebrovascular diseases due to their neuroprotective proper-ties. Studies have demonstrated the protective effect of *EGb761* on rats’ cognitive performance, involves preventing apoptosis and autophagy in models of VD, as well as improving cognitive performance in rats with VD through the activation of *AMPK-mTOR* signaling (Yin et al., 2024).

### 6.4 Berberine (BBR)

Berberine (BBR), a fundamental constituent of the Chinese herb *Rhizoma coptidis*, is an iso-quinoline alkaloid derived from *Berberidaceae* (Phogat et al., 2024). Recent research has shown that BBR possess strong anti-dysenteric qualities in addition to a variety of cardiovascular pharmacological actions, such as controlling dyslipidemia, preventing arrhythmias, inhibiting heart failure, myocardial remodeling, and lowering blood pressure. BBR prevents cardiac senescence by boosting cardiac myocytes production of KL mRNA and protein and controlling the *KL/SIRT1* signaling pathway, thereby enhancing its protective effects (Li C. et al., 2022). BBR has the ability to improve diabetic cardiomyopathy by increasing the expression of myocardial *methionine sulfoxide reductase A* (*MsrA*) and simultaneously inhibiting cardiac *CaMKII* oxidation (Sun et al., 2023). It has been investigated by Wang et al., 2023, that BBR and its derivative *tetrahydroberberrubine* (*THBru*) improve cardiac remodeling and reduce heart aging. Compared to BBR, *THBru* has a greater anti-heart aging effect because it prevents heart aging via *PHB2*-mediated mitophagy (Wang L. et al., 2023).

Moreover, researchers are progressively focused on investigating the impact of plant-derived antioxidant on heart aging. This has led to an exploration of various chemical compounds produced from plants, with the aim of promoting cardiovascular health and mitigating the adverse effects of aging on the heart. An excellent proof is seen in several naturally occurring compounds present in plants. These drugs have garnered considerable interest owing to their potential advantages for cardiovascular health, such as enhancing endothelial function, reducing inflammation, and demonstrating antioxidant qualities (Table 1). The effects outlined above are particularly relevant to the phenomenon of heart aging, as they have the potential to mitigate the oxidative stress and inflammation associated with the aging of the cardiovascular system. The research findings indicate that these antioxidants may aid in preserving optimal heart function and vascular health, perhaps decelerating the heart aging process.

**TABLE 1:** Examples of recently investigated plant-derived antioxidants associated with heart aging.

Compound	Dosage	Pathway	Impact	References
Astragalus polysaccharides	200 mg/kg/d	*SIRT-1*/*p53*	Improved *SIRT-1* protein expression in rat aortic tissue, decreased aging marker proteins, reduced hydrogen peroxide-induced cell senescence, and restored *MMP* and *T-AOC* impairment in *RAECs*.	Miao et al. (2024)
Olive leaves	100 mg/kg/d	*COX-2*/*IL-6*/*GPx*/*NOX-1*/and IL-10	Significantly reduces inflammation and oxidative stress, potentially enhancing cardiometabolic health in older patients by alleviating the metabolic and vascular changes associated with aging.	González-Hedström et al. (2021)
Garlic (*A. sativum*)	100 mg/kg^−1^/d	Na^+^/K^+−^ATPase and Ca^2+^	The experimental CRF model revealed that GE administration effectively protected the heart by lowering oxidative stress, modulating cardiac Na^+^/K^+−^ATPase activity, and regulating Ca^2+^ levels.	Gomaa et al. (2018)
EGCG	100 or 200 mg/kg/d	*cTnI*	Combat the aging-related decrease in CDD and cTnI expression.	Quan et al. (2024)
Lycopene	0.5, 1, or 2 μm/d	*SIRT1*/*Nrf2*/*HO-1*	Reduces intracellular ROS levels, the synthesis of inflammatory factors, cell adhesiveness, and the rate of apoptosis under oxidative stress conditions, therefore mitigating oxidative damage in human VECs.	Guo et al. (2023)
β-carotene	40 μM/d	*PI3K*/*Akt/mTOR*	Significantly reduced AGE-induced cell death, apoptosis, ROS production, antioxidative enzyme reduction, ER stress, autophagy, and cardioprotection in *H9c2* cells, thereby reducing ER stress and autophagy.	Zhao et al. (2020)
Chlorogenic acid	20 or 40 mg/kg/d	*Nrf2*/*HO-1*	Positive impact on vascular senescence.	Hada et al. (2020)
Chlorogenic acid	90 mg/kg/d	*AMPK*/*SIRT1*	*AMPK*/*SIRT1* pathway activation by *S1pr1* regulation decreased ISO-induced ERS and cardiac hypertrophy.	Ping et al. (2024)
Chlorogenic acid	15 mg/kg i.p./h	*Nrf2*/*HO-1*	Inhibit DT expression, activate the *Nrf2/HO-1* signaling pathway, decrease oxidative stress, and decrease apoptotic markers to lessen DOX-induced cardiotoxicity *in vivo*.	Cicek et al. (2023)
Alpha-lipoic acid	100 mg/kg/d	*Mfn1*, *Mfn2* and Foxo1 *Drp1* and *Fis1*	Preventing the aging heart against ischemia-reperfusion injury by enhancing oxidative stress, mitochondrial function, and dynamics in elderly rats.	Oskuye et al. (2024)
Bromelain	20 mg/kg/d	*AMPK*/*TFEB*	Facilitated anti-hyperlipidemic, antioxidant, and anti-inflammatory actions, contributing to the mitigation of atherosclerosis.	Chen et al. (2022a)
8-Gingerol	10 or 20 mg/kg/d	*PI3K/Akt*/*mTOR*	The findings imply that via inhibiting ROS production, apoptosis, and autophagy, ISO-induced MF may have cardioprotective benefits.	Xue et al. (2021)
Apigenin	20 μg/h	NADPH oxidase	Reduce inflammation in the PVN and down-regulating NADPH oxidase-dependent ROS generation in SHRs can improve hypertension and cardiac hypertrophy.	Gao et al. (2021)
Astaxanthin	75 mg/kg/d	*SIRT1*	Improves cardiac function and diminishes fibrosis by decreasing the phosphorylation and deacetylation of R-SMADs.	Zhang et al. (2017)
Astaxanthin	10 μg/mL/48 h	*PTP1B*/*JNK*	Reduce LPS-induced mitochondrial apoptosis in *H9C2* cells by regulating *JNK* signaling.	Xie et al. (2024)
Ferulic acid	30 mg/kg/d	*miR-499-5p*/*p21*	Protect cardiomyocytes from oxidative stress-induced injury, suggesting potential use in treating CVDs.	Sun et al. (2021)
Naringenin	100 mg/kg/d	*SIRT1*	A nutraceutical strategy utilizing NAR may ameliorate myocardial senescence by targeting essential characteristics, potentially enhancing heart function in elderly individuals.	Testai et al. (2020)
Vanillin	150 mg kg^−1^/d^−1^	*Akt/HIF-1α*/*VEGF*	Potential of Van and *PTX* in lowering MI through improving cardiac angiogenesis and controlling apoptosis, inflammation, and oxidative stress.	Elseweidy et al. (2023)
Chrysin	100 mg/kg/d	*eNOS* and *Nrf2*	Prevents myocardial complications from hypercholesterolemia-induced oxidative stress by activating eNOS and *Nrf2* signaling.	Yuvaraj et al. (2021)
Cinnamaldehyde	45 and 90 mg/kg/d	*NLRP3*	Exhibits cardioprotective characteristics by suppressing *NLRP3* inflammasome activation and GSDMD-mediated pyroptosis in cardiomyocytes, presenting potential uses for myocardial ischemia/reperfusion damage.	Luan et al. (2022)
Pterostilbene	20 mg kg^−1^ day^−1^	*AMPK*/*Nrf2*/*HO-1*	In diabetic rats, it decreases inflammation and heart oxidative stress.	Kosuru et al. (2018b)
Caffeic Acid Phenethyl Ester	10 mg/kg i.p./d	*Sirt6/Nrf2*	Effectively suppresses oxidative stress and promotes protective polarization in microglia.	Wang et al. (2023b)
Caffeic acid derivative	3 mg/kg/i.p./d	*TGF-β*/*SMAD*/*NOX4*	Potential to prevent the progression of Ang II-induced cardiac remodeling.	Lee et al. (2023)
Caffeic acid phenethyl ester	1 mg/kg/d	*SIRT1*/*eNOS*/*NF-κB*	The treatment improved MIRI by reducing oxidative stress, inflammatory response, fibrosis, and necrocytosis.	Li et al. (2018)
Fisetin	20 mg/kg/d	*SIRT1*/*Nrf2*	Effectively treats DOX-induced cardiomyopathy by inhibiting ferroptosis.	Li et al. (2022b)
Hesperidin	25–50 mg/kg/d	*Sirt1*/*Nrf2*	Protects against ISO-induced myocardial ischemia by regulating oxidative stress, inflammation, and apoptosis.	Liu et al. (2021)
Jin-Xin-Kang	4.38–13.14 g/kg/d	*CaN*/*Drp1*	Plays a crucial role in cardioprotection, particularly in regulating mitochondrial function.	Lin et al. (2024)
Genistein	100 mg/kg/d	*miR-451*/*TIMP2*	Promoted the expression of *miR-451* and inhibited cardiac hypertrophy.	Gan et al. (2019)
Saponins	10–30 mg/kg	*AMPK*/*mTOR*/*ULK1*	Reduce in basal autophagy in cardiomyocytes and enhance the prevention of aging-related cardiac dysfunction.	Huang et al. (2023)
*Salvia haenkei*	0.5 mg/kg^−1^/d	*p16–CDK6*	Increases the lifespan of mice via controlling cellular senescence and interfering with the *p16-CDK6* association.	Zumerle et al. (2024)
Baicalin	5 mg/kg/d	*AMPK/mTOR*	Cardiomyocytes apoptosis and autophagy in response to Ang II were reduced by the inhibition of the *AMPK/mTOR* pathway.	Cheng et al. (2024)

## 7 Future prospective and conclusions

The rising prevalence of age-related heart aging underscores the urgent need for effective treatment strategies. In this context, investigating plant-derived antioxidants shows considerable potential in addressing this challenge. The results presented in this review confirm that some plant-based antioxidants such as polyphenols, terpenoids and alkaloids might reduce heart aging effects and moderate expression of genes involved in the same process. Plant-derived antioxidants provide protection to the heart by acting on several molecular pathways and signaling cascades. It will need further study to fully comprehend the complex interplay between antioxidant chemicals produced from plants and the aging process in the cardiovascular system, with a particular emphasis on the examination of pathways and signaling cascades. To enhance the effectiveness of antioxidant treatments, it is crucial to evaluate gene expression patterns, epigenetic modifications, and cellular signaling networks. With the use of innovative techniques and formulations, such as targeted-tissue delivery and controlled-release formulations, it is possible to overcome obstacles like poor stability and enhance the absorption and distribution of these antioxidants. The increasing prevalence of aging-related cardiovascular disorders necessitates the development of effective treatment approaches.

This review highlights the potential of plant-derived antioxidants, including polyphenols, terpenoids, and alkaloids, to mitigate the negative effects of heart aging and alter gene expression. These compounds offer cardioprotective benefits through various molecular pathways and signaling cascades, highlighting the need for efficient treatment approaches in this rapidly aging-related issue. The development of innovative delivery methods and formulations can significantly improve the therapeutic effectiveness of plant-based antioxidants. These methods can overcome challenges like poor solubility, low stability, and restricted tissue targeting associated with some plant-based agents, such as transdermal patches, controlled release formulation, and nanoparticle-based drug delivery. Effective collaboration among researchers in the fields of plant biochemistry, pharmacology, molecular biology, and cardiovascular medicine is essential for converting promising preclinical research findings into viable therapeutic interventions. For plant-base antioxidants to be used in comprehensive heart aging care management, rigorous clinical trials assessing their safety, effectiveness, and long-term effects on heart aging and related biomarkers are necessary to determine their therapeutic potential.

In conclusion, plant antioxidants, found in fruits, vegetables, and other plants, have been found to have significant cardioprotective properties. These compounds, including carotenoids, phenolic acids, and flavonoids, work by neutralizing ROS, enhancing antioxidant capacity, and modulating cellular signaling pathways. Plant antioxidants not only prevent cardiovascular damage but also promote tissue repair and functional recovery. They may be effective adjuncts in treating CVDs, either in the diet or as supplements. However, further research is needed to optimize doses, conduct clinical trials, evaluate bioavailability, metabolism, and specific effects of each antioxidant, analyze interactions with other drugs or treatments, identify potential side effects from high doses, and identify potential side effects of their use in treating CVDs. Researchers may discover novel approaches to prevent and manage heart aging by using the wide range of plant-based antioxidants and their complex mechanisms of action. This might eventually result in improved quality of life and health outcomes for the aging population.
